# Association of a High Healthy Eating Index Diet with Long-Term Visceral Fat Loss in a Large Longitudinal Study

**DOI:** 10.3390/nu16040534

**Published:** 2024-02-14

**Authors:** Sunmin Park

**Affiliations:** Department of Food and Nutrition, Obesity/Diabetes Research Center, Hoseo University, Asan-Si 31499, Republic of Korea; smpark@hoseo.edu; Tel.: +82-41-540-5345; Fax: 82-41-548-0670

**Keywords:** waist circumference reduction, healthy eating index, Korean balanced diet, prediction model, dietary pattern, abdominal obesity

## Abstract

We aimed to investigate the association of a sustainable diet with a long-term reduction in waist circumference (WC) while identifying novel biomarkers for WC reduction (WCR). The participants were recruited initially during 2004–2013 in a large hospital-based cohort, and the follow-up measurements were conducted during 2012–2016. The 65,611 adults aged 45–75 were categorized into WC-loss (*n* = 22,290) and WC-gain (*n* = 43,321). Each study investigated demographic, anthropometric, biochemical, genetic, and dietary factors. The modified Healthy Eating Index (MHEI), dietary patterns, and glycemic index were calculated from a validated semi-quantitative food frequency questionnaire. Novel biomarkers influencing WC reduction were identified using machine learning approaches. A WCR was inversely associated with metabolic syndrome (MetS) risk and its components. Daily energy intake did not differ between those with and without WCR. However, MHEI, which represents diet quality, demonstrated a positive association with WCR. Among various dietary patterns, the Asian-style balanced diet (ABD), including more fermented soybeans and less restricted salt than the Diet Approach to Stop Hypertension, was positively associated with WCR. However, an inverse association was observed between the diet that was high in noodle and processed meat consumption and that which was high in rice consumption. However, the PRS for abdominal obesity did not significantly interrupt WCR. The receiver operating characteristic curve in the prediction model for WCR was about 0.86. The biomarkers in the models included MetS components, inflammation index, diet components, alcohol consumption, and smoking status, but not genetic factors. In conclusion, adopting a high-quality diet with a high MHEI like ABD leads to WCR, irrespective of genetic influences. These results could be applied to develop effective strategies for preventing and managing abdominal obesity.

## 1. Introduction

The escalating global prevalence of obesity has been a health challenge over the last three decades, including among Asian populations [1]. Among the multifaceted dimensions of obesity, abdominal obesity stands out as a critical concern due to its association with excess visceral fat accumulation around the abdominal organs [2]. Unlike subcutaneous fat, visceral fat is metabolically active, significantly influencing various physiological processes and contributing to insulin resistance, inflammation, and dyslipidemia [2]. It heightens susceptibility to chronic diseases such as cardiovascular disease (CVD), type 2 diabetes (T2D), and certain cancers [2]. Identifying factors that facilitate sustained reduction in abdominal obesity is paramount for public health initiatives and formulating effective strategies to manage body weight and prevent obesity-related metabolic diseases.

While advanced imaging techniques like computed tomography (CT) and magnetic resonance imaging (MRI) accurately assess visceral fat distribution, the measurement of waist circumference (WC) has emerged as an inexpensive yet reliable indicator of visceral fat accumulation. WC can be used to screen and monitor abdominal obesity [3] and can be a robust predictor of abdominal obesity and health risks. Indeed, various global health organizations have used the measurement of WC to define abdominal obesity. Since it is simple, cost-effective, and non-invasive, WC measurement is useful in clinical settings and large cohort studies [3].

The Mediterranean diet, a healthy eating pattern characterized by a plant-based diet that is high in unsaturated fat and lowers the risk of chronic metabolic diseases, has received significant attention. Studies such as the Prevention with Mediterranean Diet (PREDIMED) trial have demonstrated better outcomes regarding cardiovascular and other chronic diseases in individuals at high cardiovascular risk who are on the Mediterranean diet [4]. However, adaptability, sustainability, and compliance related to specific diets, such as the Dietary Approaches to Stop Hypertension (DASH) and Mediterranean diets, for reducing abdominal obesity in regions like Asia pose challenges. This highlights the need to explore region-specific dietary patterns for sustained reductions in WC. Whereas cross-sectional studies have explored associations between WC and diets [5,6], only a few longitudinal studies have investigated the direct impact of dietary patterns or diet quality on reducing WC [7].

Moreover, the intricate interplay between genetic factors and abdominal obesity remains a relatively unexplored domain, especially in large longitudinal studies. The Asian-style balanced diet (ABD), similar to the modified DASH and other healthy diets, has been associated with a lower WC in large cross-sectional studies and randomized clinical trials [8,9]. However, its potential role in reducing abdominal obesity remains to be elucidated while considering the role of genetic and other environmental factors. Therefore, we comprehensively investigated the efficacy of a healthy, sustainable diet in reducing waist circumference (WC) and identified novel biomarkers for WC reduction in a large longitudinal cohort study. This study determined the role of genetic, biochemical, and lifestyle factors, including dietary patterns and diet quality, in sustained WC reductions over approximately 5 years. Additionally, we aimed to innovate the methods to determine the biomarkers for WC reduction by implementing machine learning (ML) techniques to integrate other diverse factors. The integrated approach promises to enhance our understanding of the complex interplay of various factors that play a role in reducing abdominal obesity, thus providing valuable insights for effective public health strategies and personalized interventions.

## 2. Methods

### 2.1. Setting and Recruitment

The study enrolled Korean adults aged over 40 years who initially participated voluntarily in the large city-based hospital cohort of the Korean Genome and Epidemiology Study (KoGES) conducted by the Korea Disease Control and Prevention Agency (KDCA, formerly Korea Centers for Disease Control and Prevention) from 2004 to 2013. Follow-up recruitment was conducted from 2012 to 2016. A total of 67,734 individuals participated in both recruitments. Participants with cancer, thyroid disorder, chronic kidney disease, and brain-related diseases that could influence energy metabolism (*n* = 2123) were excluded. The final number of participants included in the present study was 65,611: men comprised 34.0% (*n* = 22,290), and their average age was 61.6 ± 0.07, whereas women comprised 66% (*n* = 43,321), and their average age was 57.2 ± 0.04. Due to gender differences, the participants were categorized by gender. Experimental procedures for the first and second (follow-up) recruitments in the city-based hospital cohort were conducted under the Declaration of Helsinki. They were approved by the Institutional Review Board of the Korean National Institute of Health (KBP-2015-055) and Hoseo University (1041231-190902-BR-099-01) on 2 September 2019. All participants provided written informed consent.

### 2.2. Definition of Abdominal Obesity Based on Waist Circumference

Abdominal obesity was defined using a WC cutoff of 90 cm for men and 85 cm for women [5]. Waist circumferences were measured using a flexible measuring tape around the belly button, the highest part of the waist, ensuring a snug but not tight fit [10]. Participants were stratified into two categories: a normal WC (*n* = 51,640 and 50,674 at the first and second recruitments, respectively) and a high WC, indicative of abdominal obesity (*n* = 13,971 and 14,937 at the first and second recruitments, respectively). The modulation of abdominal obesity, as assessed by changes in WC, was calculated by subtracting the WC at the follow-up measurement from the first measurement. The WC change, indicating abdominal obesity modulation, was further categorized into two groups based on a zero cutoff (WC-loss, *n* = 32,197; WC-gain, *n* = 33,414) or quintiles using the 20th percentiles.

### 2.3. Demographic, Anthropometric, and Biochemical Measurements

The detailed data were gathered through interviews conducted by a trained technician during the first and second recruitment phases [10]. The residence area was defined as the place of residence in a specific province for over six months. The participants provided information on age, gender, living area, education, income, job, alcohol consumption, smoking status, and regular exercise undertaken, following a previously established questionnaire format. Monthly household income, education, and employment for over six months were categorized according to the specific outlines in previous publications [10]. The daily alcohol intake was computed by multiplying the frequency of alcohol consumption by the quantity consumed on each occasion. The smoking status was categorized into three groups: current smokers, former smokers, and never smokers. Current smoking was determined by whether participants had smoked more than 100 cigarettes at any point in their lifetime. In contrast, former smokers were individuals who had not smoked in the last six months [10]. Regular exercise was defined as more than 30 min of moderate physical activity three or more days per week [11].

Standard protocols were employed for assessing body weight, height, and WC [11]. The body mass index (BMI) was calculated as the ratio of body weight (kg) to height squared (m^2^). Blood pressure was measured on the right arm after at least 20 min of rest. During measurement, the individual was seated in a chair with the feet on the floor and the arm supported so that the elbow was at the level of the heart. Systolic (SBP) and diastolic (DBP) blood pressure were recorded for analysis. Biochemical measurements were conducted using plasma and serum obtained after fasting for over 12 h [11]. Lipid profiles including low-density lipoprotein cholesterol (LDL-C), high-density lipoprotein cholesterol (HDL-C), triglycerides (TG), glucose, glutamate oxaloacetate transaminase (GOT), glutamate pyruvate transaminase (GPT), γ-glutamyl transpeptidase (γ-GTP), blood urea nitrogen (BUN), and creatinine concentrations in the plasma or serum were determined using a Hitachi 7600 Automatic Analyzer (Hitachi LTD., Tokyo, Japan). White blood cell (WBC) counts and hemoglobin A1c (HbA1c) levels in heparin-treated blood were determined using the same analyzer. Serum high-sensitivity C-reactive protein (hs-CRP), fibrinogen, and platelet concentrations were measured using an enzyme-linked immunosorbent assay (ELISA) kit. Morning urine samples were collected to assess pH, protein, glucose, and blood levels.

### 2.4. Food and Nutrient Intake Assessments, Dietary Patterns, and Dietary Inflammatory Index

The semi-quantitative food frequency questionnaire (SQFFQ) was developed and validated for the KoGES, including 106 food items commonly consumed by Koreans [11]. Participants reported their regular food intake over the past 12 months to a skilled technician, detailing the frequency and portion size of each item. The food intake of each item was calculated by multiplying its frequency of consumption by the portion size as indicated by a picture. After SQFFQ completion, the daily food intake was determined by summing the intakes of 106 food items. Nutrient intakes were computed using the computer-aided nutritional analysis program 3.0 (CAN-Pro), a nutrient database program developed by the Korean Nutrition Society (KNS) [11]. Each nutrient intake was compared to dietary reference intake [11].

For dietary pattern analysis, the 29 food groups derived from the SQFFQ were treated as independent variables in a factor analysis using the FACTOR procedure. This analysis utilized eigenvalues >1.5 and an orthogonal rotation procedure (Varimax) [12]. Dietary factor-loading values ≥0.40 indicated significant contributions to the identified dietary patterns. The participants’ diets were categorized into ABD, plant-based diet (PBD), a diet with high fast-food consumption (FFD), and a diet with high rice consumption (HRD). The ABD included fermented soybeans, fish, seafood, meat, seaweed, vegetables, kimchi, and mushrooms, similar to the modified Diet Approach to Stop Hypertension (M-DASH). However, ABD included more fermented beans and a less-restricted salt intake (about 6 g/day) than M-DASH. In contrast, the PBD included elevated levels of eggs, milk, beverages, fruits, and nuts, whereas the FFD was rich in noodles, soups, meats, processed meats, and fast foods. The HRD was mainly composed of rice.

The dietary inflammatory index (DII) was calculated by multiplying the dietary inflammatory weights of 38 food and nutrient components based on the daily intakes. The sum of the scores for these 38 items was divided by 100. Dietary inflammatory weights, reported in a previous study [13], guided this calculation. Garlic, ginger, saffron, and turmeric were excluded from the DII equation as these data were not included in the SQFFQ.

### 2.5. Modified Healthy Eating Index (MHEI) Definition

The KDCA has adapted the HEI for Koreans, resulting in a modified version known as MHEI. The MHEI is designed to comprehensively assess diet quality, particularly emphasizing its effectiveness in evaluating the quality of Asian diets. The index is based on assessing the adequacy of food intake, the appropriateness of saturated fat, sugar, and sodium intake, and the balance of energy, fat, and carbohydrate intake [14]. The MHEI is composed of the adequacy (8 items), moderation (3 items), and balance (3 items) domains of energy intake, and the scores of each item in these domains have been described in an earlier study [15,16]. The MHEI scores are assigned using data from the Dietary Guidelines for Korean Adults and the Dietary Reference Intake (DRI) for Koreans in 2015 [17]. The MHEI scores for this study were calculated using the SQFFQ. The maximum scores for each item in the modified MHEI are provided in Supplementary Table S1. Each item was given a score, with a possible total score of between 0 and 150. Higher MHEI scores indicated healthier diets. The modified MHEI added several items, namely seaweed, fish, beans (including fermented bean sauce), and nuts in the adequacy domain, and the percentage of energy from fast foods, sweets, sugar beverages, and noodles in the moderation items. There was no modification in the balance of energy intake. The standards for additional items were assigned based on the recommendations of the World Health Organization (WHO), Food and Agriculture Organization (FAO), and Korean dietary guidelines.

### 2.6. Genetic Factors Related to the Risk of Abdominal Obesity

The Center for Genome Science at the Korea National Institute of Health (KNIH) conducted a single nucleotide polymorphism (SNP) analysis on the city-hospital-based cohort belonging to the KoGES. The genomic DNA from each participant was extracted from their whole blood, and the genotypes were measured using a Korean Chip (Affymetrix, Santa Clara, CA, USA) designed to include the disease-related SNPs [18]. The genotyping accuracy was determined using Bayesian Robust Linear Modeling using the Mahalanobis Distance Genotyping Algorithm (BRLMM) [19]. The genotyping accuracy, missing call rate of genotypes, and heterozygosity were greater than 98%, <4%, and <30%, respectively, and there was no gender bias. The genetic variants also met the Hardy–Weinberg equilibrium (HWE; *p* > 0.05) and the minor allele frequency (MAF; >1%) criterion [19].

The genetic variants associated with abdominal obesity were identified using the data of the hospital-based KoGES cohort. The best set of genetic variants with interactions influencing abdominal obesity was generated with a sign rank test (*p* < 0.05) of trained balanced accuracy (TRBA) and testing balanced accuracy (TEBA), adjusting for the covariates by generalized multifactor dimensionality reduction (GMDR) [20]. Ten-fold cross-validation (CV) was used to estimate the CV consistency (CVC), because the sample size was larger than 1000 [21]. Nine to ten out of ten met the perfect cross-validation criteria with regard to CVC. The genetic variants for abdominal obesity selected from GMDR in each set were used to calculate the polygenic risk score (PRS) by summing the number of risk alleles of each SNP in the selected best model [22].

### 2.7. Experimental Design for the Machine Learning (ML) Approach for Predicting WC Reduction

As shown in an earlier study [23], we leveraged 220 genetic, demographic, and environmental variables as potential predictors after excluding some correlated variables. Handling missing values involved imputing the mode for categorical variables and the mean for continuous variables. Normalization of each continuous variable to the z-score was implemented to maintain consistency across the dataset. To evaluate the model performance, we allocated 10% of the data for a validation set, and then the remaining 90% was utilized for training and testing at an 8:2 ratio. The dataset was further standardized, resulting in 220 variables, and training and test sets were generated using the randomized grid search method. We selected appropriate models to optimize predictive accuracy, the area under the receiver operating characteristic curve (AUROC) curve, and the k-fold in the test dataset. The algorithmic models for predicting metabolic status were chosen with logistic regression, extreme gradient boosting (XGBoost), random forest, and a deep neural network (DNN). For the DNN, the iterations were performed with specific parameters: 25 epochs, a 0.1 validation split, and 100 layers. These adjustments enhanced the model’s capacity to accurately predict reductions in WC by integrating genetic, demographic, and environmental variables.

### 2.8. Prediction Model for WC Reduction

Upon analyzing the 220 variables, we calculated the relative importance of values extracted from the random forest, XGBoost, and DNN algorithm models to pinpoint the genetic model within the training set. The optimal model for predicting decreases in WC was determined based on superior metrics such as the highest AUROC, accuracy, and k-fold in the test dataset, chosen from the random forest, XGBoost, and DNN algorithm models. Despite a comprehensive evaluation, the algorithmic models did not reveal a clear positive or negative relationship. To unravel the decision-making processes within these models, we employed the SHapley Additive exPlanation (SHAP) method (accessible at https://shap.readthedocs.io/en/latest/index.html, accessed on 23 July 2023). SHAP was an instrumental tool that, in detail, explained the selected models derived from the random forest and XGBoost algorithms. We selected three prediction models generated by the XGBoost, DNN, and random forest algorithms to extend our predictions to the risk of WC increase within a city-hospital-based cohort.

### 2.9. Statistical Analysis

The sample size for the study was calculated using the Gpower calculator (Gpower 3 Software, the University of Dusseldorf, Dusseldorf, Germany). The number of participants required was more than 50,000 to achieve significance at α = 0.05 and β = 0.99, at an odds ratio of 1.05. The total number of participants, 65,611, was sufficient to meet the significance goal.

Statistical analyses were carried out using SAS version 9.3 (SAS Institute, Cary, NC, USA). Missing data were imputed with means for continuous variables and mode for categorical variables only in machine learning analysis. Other analyses were conducted using the available data. Descriptive statistics for categorical variables, such as gender, education, and smoking status, were examined by analyzing the frequency distributions within the WC-loss and WC-gain groups stratified by gender. Significant differences were evaluated using the Chi-squared test. For continuous variables, descriptive values, expressed as means and standard deviations, were delineated across the WC-loss and WC-gain groups and gender categories. Adjustments were made for age, education, income, residence area, follow-up time, daily energy intake, exercise, smoking status, and alcohol consumption. The differences were assessed using a two-way analysis of variance (ANOVA) with an interaction term to accommodate covariate adjustments, including age, residence region, education, income, follow-up duration, energy intake, drinking status, smoking status, and exercise. Subsequently, post-multiple comparisons among the gender and WC-loss groups were executed using Tukey’s test. Adjusted odds ratios (OR) and 95% confidence intervals (CI) were computed for the highest WC-gain group as the reference. The covariates included age, residence region, education, income, follow-up duration, daily energy intake, drinking status, smoking status, and exercise. The statistical approach enabled a thorough examination of associations while accounting for relevant covariates.

Prediction models were developed utilizing the Scikit-learn and TensorFlow platform in Python 3.8.5 (available at https://www.python.org/downloads/windows/, accessed on 20 August 2023). The WC reduction prediction models were created by employing various algorithms, including logistic regression, XGBoost, and random forest, utilizing Scikit-learn in Python 3.8.5. Additionally, the DNN prediction model was generated using the TensorFlow platform.

## 3. Results

### 3.1. Characteristics of the Participants in the Follow-Up Study

The participants in the WC-loss group had higher BMI scores and waist circumferences than those in the WC-gain group in the first recruitment (Supplementary Figure S1). In the follow-up study, the participants with a decrease in WC were older than those with an increase in WC for both genders (Table 1). The number of female participants was higher in the WC-gain group than in the WC-loss group, but no such significant differences were observed in males. Education affected the reduction in WC only in women, and the reduction percentage was lower in those with a college education or higher (Table 1).

### 3.2. Association of WC Reduction with Metabolic Syndrome (MetS) and Its Components at the Follow-Up Study

Over 5 years, the WC-loss group showed a WC decrease of about 5 cm compared to the WC-gain group across both genders, indicating that the decrease in WC in the WC-loss group was about 1 cm per year (Table 1). Consistent with WC, BMI was also lower in the WC-loss group than in the WC-gain group. The WC-loss group had a lower incidence of MetS than the WC-gain group across both genders (Table 1). Among the MetS components, total cholesterol, HDL-C and LDL-C concentrations, SBP, and DBP were lower in the WC-loss group than in the WC-gain group (Table 1). Interestingly, serum glucose and HbA1c concentrations did not vary significantly between the WC-loss and WC-gain groups (Table 1). The incidence of myocardial infarction and stroke did not significantly differ with gender or WC reduction. However, the incidence of combined myocardial infarction and stroke was lower in the WC-loss group than in the WC-gain group, suggesting that the persons having cardiovascular events were more likely to have gained body weight (Table 1).

**Figure 1:**
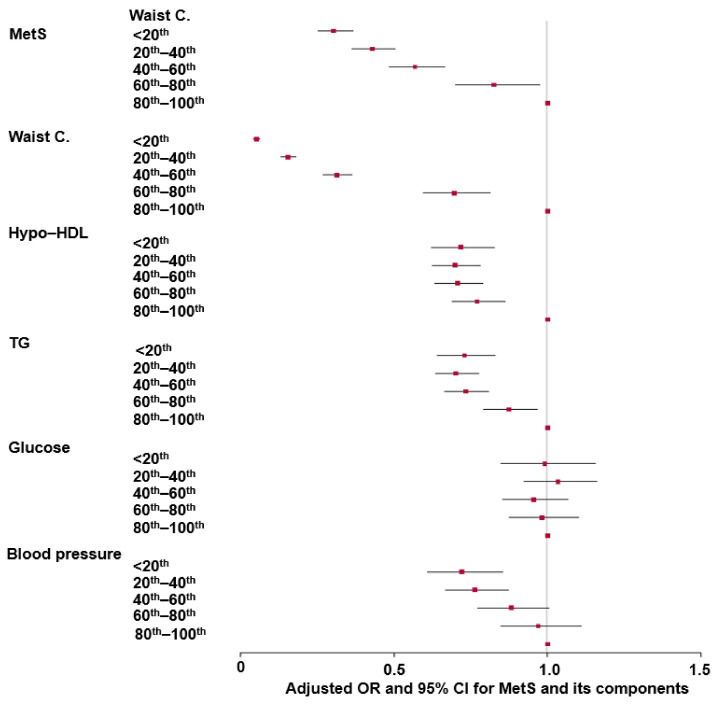
Adjusted odds ratios (red square) and 95% confidence intervals (line) for metabolic syndrome and its components based on waist circumference (WC) changes. Waist circumferences were divided into five groups by quintiles, and the highest group (80th–100th percentile) was used as the reference group in a logistic regression adjusting for covariates, including age, residence region, education, income, follow-up duration, energy intake, drinking status, smoking status, and exercise. MetS, metabolic syndrome; C., circumference; TG, triglyceride.

The WCs were divided into five groups by quintiles. The highest quintile group (the highest WC-gain group) was the reference group (Figure 1). When the reduction in WC was greater, the adjusted OR for MetS was lower, suggesting that the participants belonging to the lowest quintile exhibited about 0.4 times lower MetS risk than those in the highest quintile. Similar to MetS, hypo-LDL, TG, and blood pressure also showed a decrease in the adjusted OR, with a more significant reduction in WC when compared with the highest quintile group (Figure 1). However, the changes in serum LDL, TG, and blood pressure were smaller than of the changes in MetS. Serum glucose concentrations were not associated with decreased WC (Figure 1).

### 3.3. Effects of Dietary Nutrients on WC Reduction in the Follow-Up Study

Average energy intake was less than the estimated energy requirement (EER) in all groups and did not vary between the WC-loss and the WC-gain groups for either gender (Table 2). The intake of carbohydrates; fat; saturated, monounsaturated, and polyunsaturated fatty acids; proteins; fiber; calcium; and sodium showed gender differences. However, these did not differ according to gender between the WC-loss and the WC-gain groups (Table 2). The intake of vitamins C and D and flavonoids did not vary between the WC-loss and the WC-gain groups for either gender (Table 2). Glycemic index scores were lower in the WC-loss group than in the WC-gain group. Coffee and alcohol consumption was lower in the WC-loss group than in the WC-gain group (Table 2). The WC-loss group had a higher percentage of participants engaging in exercise than those not exercising for both genders, but the percentage of smokers was lower only among men. (Table 2).

**Figure 2:**
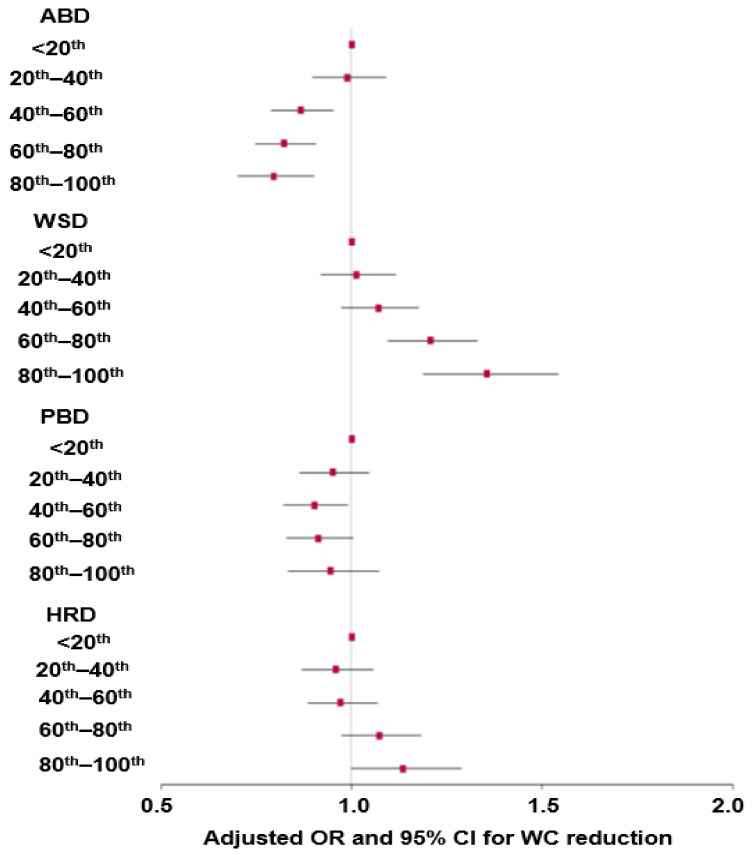
Adjusted odds ratios (red square) and 95% confidence intervals (line) for waist circumference (WC) reduction based on the quintiles of each different dietary pattern score. The scores of each dietary pattern were divided into five groups by quintiles, and the lowest intake group was used as the reference group in a logistic regression adjusting for covariates, including age, residence region, education, income, follow-up duration, energy intake, drinking status, smoking status, and exercise. ABD, Asian-style balanced diet; WSD, Western-style balanced diet; PBD, plant-based balanced diet; HRD, diet with high rice consumption.

Interestingly, a higher proportion of the participants in the WC-loss group were on an M-DASH, basically ABD, than those in the WC-gain group for both genders. However, the same was true of PBD, though only in men (Table 2). A lower proportion of the participants in the WC-loss group consumed the FFD and HRD than those in the WC-gain group. WC reduction was closely associated with dietary patterns (Table 2). The reduction in WC was categorized into quintiles; the lowest loss (the highest WC gain) was used as the reference (Figure 2). A higher intake of the M-DASH was positively associated with a 1.3-fold reduction in WC. However, while WC reduction appeared to be positively associated with a high intake of PBD, the association was not significant (Figure 2). Higher intake of the FFD and HRD were negatively associated with a 0.75- and 0.92-fold reduction in WC (Figure 2).

### 3.4. Impact of MHEI Scores on Decreasing WC in the Follow-Up Study

**Figure 3:**
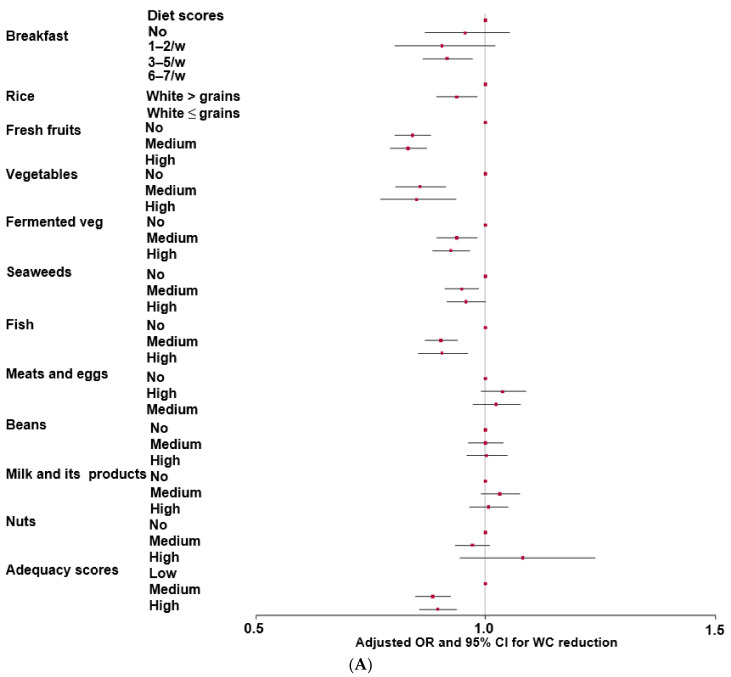

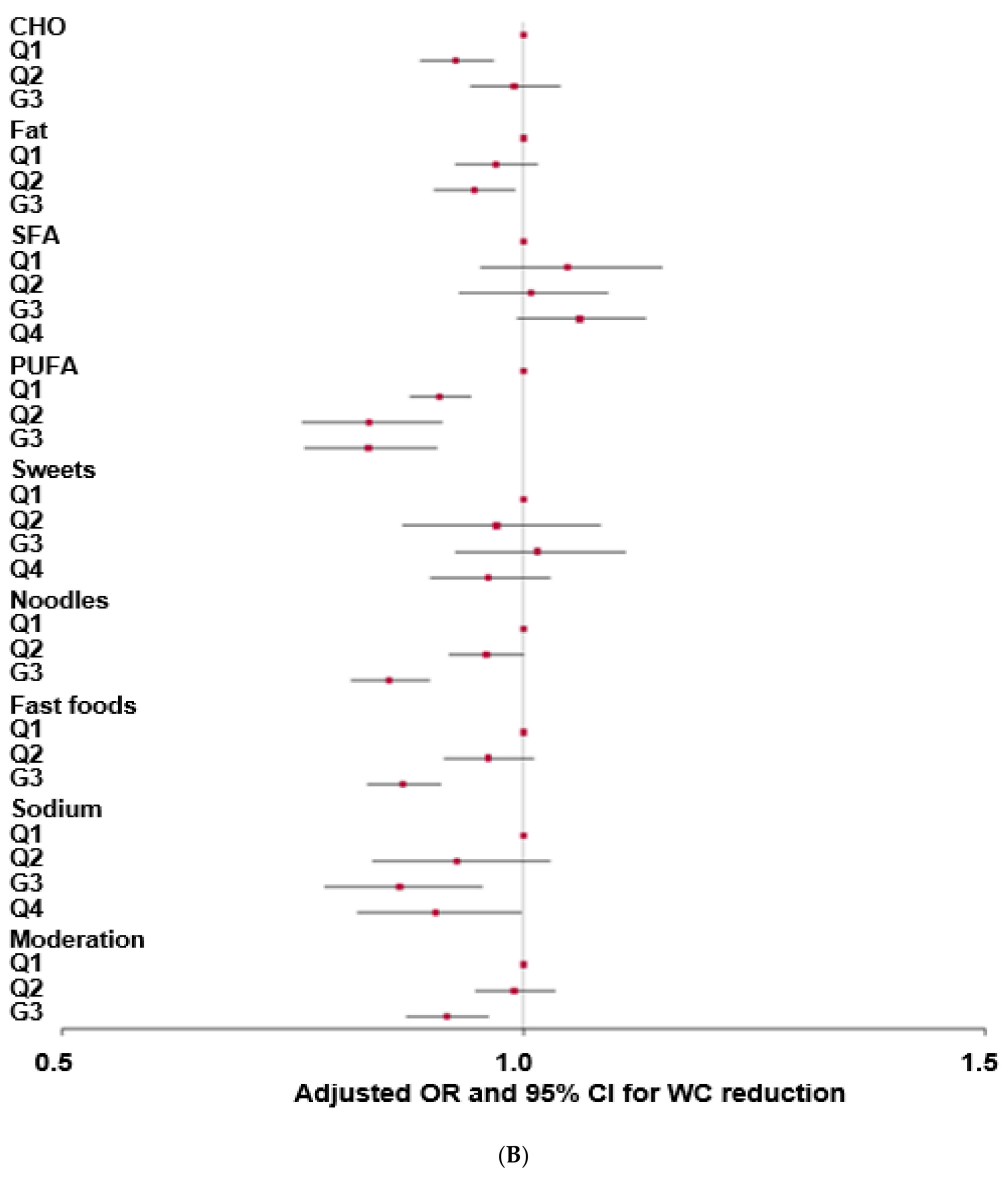

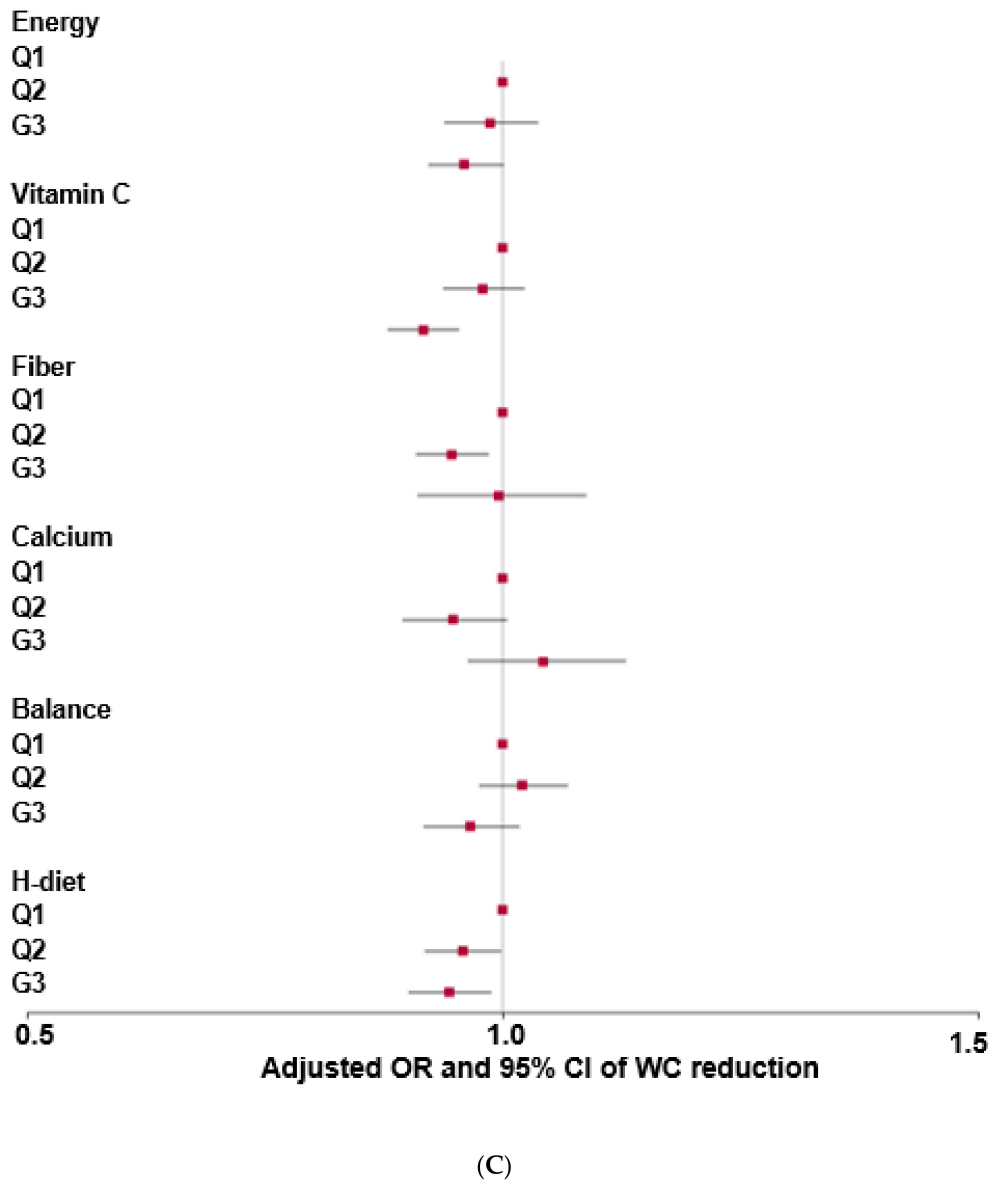
Odds ratios (red square) and 95% confidence intervals (line) for the modified healthy eating index (MHEI) based on waist circumference (WC) reduction after adjusting for covariates including age, residence region, education, income, follow-up duration, energy intake, drinking status, smoking status, and exercise. (**A**) Adequacy scores; (**B**) moderation scores; (**C**) balance scores and total MHEI scores. CHO, carbohydrate intake; SFA, saturated fatty acids; PUFA, polyunsaturated fatty acids. Q1, Q2, Q3: tertiles.

The MHEI is composed of the adequacy, moderation, and balance components of nutrient and food intake. There were gender differences in all MHEI scores except for energy, saturated fat, and fast foods (Table 3). The components of the adequacy scores, except for the scores of meats and eggs, milk and its products, and nuts, were significantly higher in the WC-loss than the WC-gain groups, suggesting that participants with a higher adequacy score showed a greater decrease in WC than those with a lower score (Table 3). Moreover, the total adequacy score was higher in the WC-loss group than in the WC-gain group. The total adequacy score was inversely associated with WC change, with WC gain as the reference (Figure 3A). For the adequacy components, a regular breakfast with grains and a higher intake of vegetables, fermented vegetables, fruits, seaweeds, and fish were inversely associated with WC gain. Therefore, participants consuming an adequate intake of healthy foods showed decreased WC. The overall moderation score was higher in the WC-loss group than in the WC-gain group. Only the moderation scores for fast food and noodles affected WC, with higher intakes in the WC-gain group than in the WC-loss group (Table 3). Among the moderation score components, the scores for carbohydrates, polyunsaturated fatty acids (PUFA), noodles, fast foods, and sodium were inversely associated with WC reduction (Figure 3B). Therefore, a lower intake of fast foods and noodles was associated with greater decreases in WC.

The total scores for the balance category were not significantly different between the two groups (Table 3). In the balance category, vitamin C intake was higher in the WC-loss group than in the WC-gain group. Vitamin C was inversely associated with WC gain (Figure 3C). The total MHEI scores for adequacy, moderation, and balance were significantly higher in the WC-loss group than in the WC-gain group for both genders (*p* < 0.05) (Table 3). The overall MHEI score was inversely associated with WC gain (Figure 3C).

### 3.5. Genetic Factors

**Figure 4:**
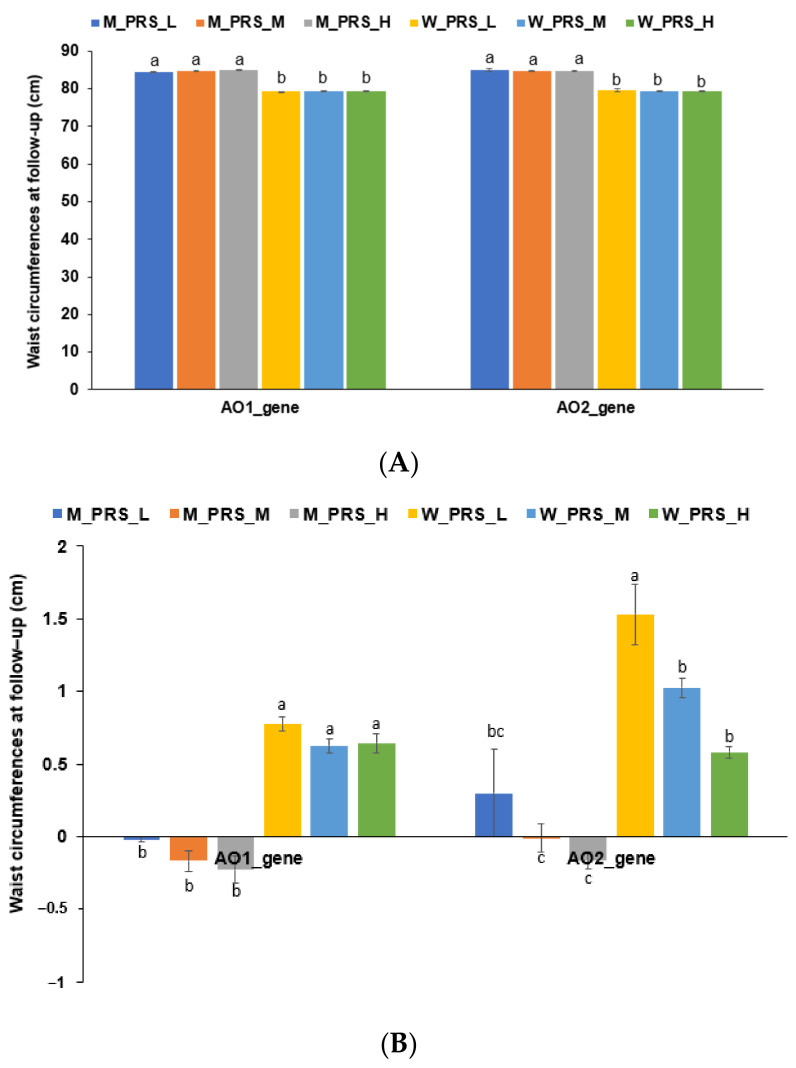
Waist circumference (WC) at the follow-up measurement according to two different sets of polygenic risk scores (PRS) for waist circumference determined from the initial recruitment data. (**A**) WC according to PRS and gender; (**B**) WC changes according to PRS and gender. ^a,b,c^ Different letters indicate significant differences among the groups in Tukey’s test results at *p* < 0.05. AO, abdominal obesity; M, men; W, women.

An analysis of the genetic variants associated with abdominal obesity based on WC cutoff was conducted on the first recruitment stage of the city-hospital-based cohort of our previous study [22]. *COL3A1*_rs3106801, *ADAMTS3*_rs13105983, *KCNQ5*_rs2796044, *ZFHX3*_rs9938769, and *MIR17HG*_rs7318578 were selected after adjusting for covariates including age, gender, residence area, education, income, energy and alcohol intake, exercise, smoking status, and LBM. The five genetic variants were used to generate PRS models (AO1_PRS). WC did not differ among the AO1_PRS groups for either gender during the follow-up measurement. However, it was much lower in men than women (Figure 4A). The reductions in WC were not significantly different among the AO1_PRS groups for either gender (Figure 4B). The other genetic variant set was selected and adjusted with the same covariates, excluding LBM. The following set of genetic variants were selected: *SEC16B*_rs543874, *KCNQ5*_rs2796052, *CDKAL1*_rs9356744, *BDNF*_rs6265, *FTO*_rs1421085, *MC4R*_rs17782313, and *GIPR*_rs1444988703. Like the AO1_PRS, initial WC did not vary among the PRSs of the AO2_PRS groups for either gender. Unlike AO1_PRS, there was a higher gain in WC in the low AO2_PRS group than in the medium and high AO2_PRS groups at the follow-up measurement (Figure 4B). These results suggest that, unlike AO2_PRS, high AO1_PRS marginally inhibits decreases in WC. Therefore, individuals with the risk alleles of common genetic variants for abdominal obesity can reduce their waist circumference through lifestyle modifications.

### 3.6. Prediction of Biomarkers Associated with WC Reductions Using the ML Approach during the Follow-Up Study

**Figure 5:**
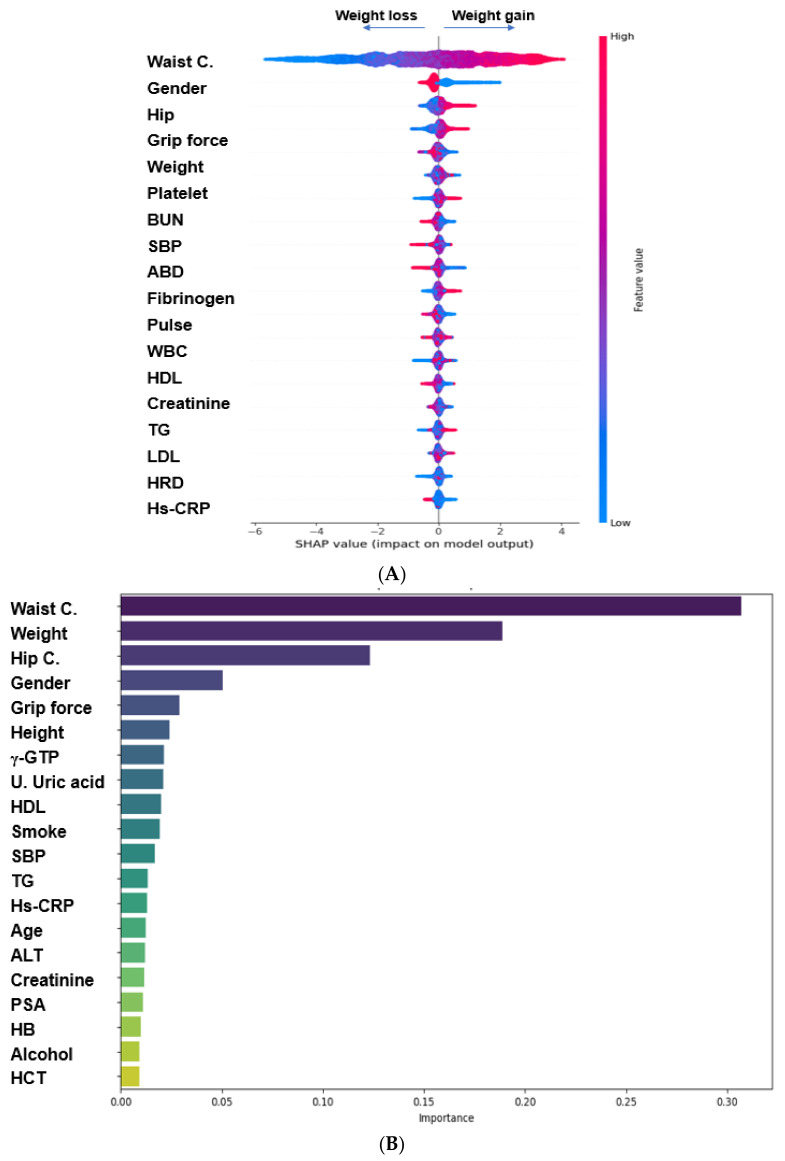

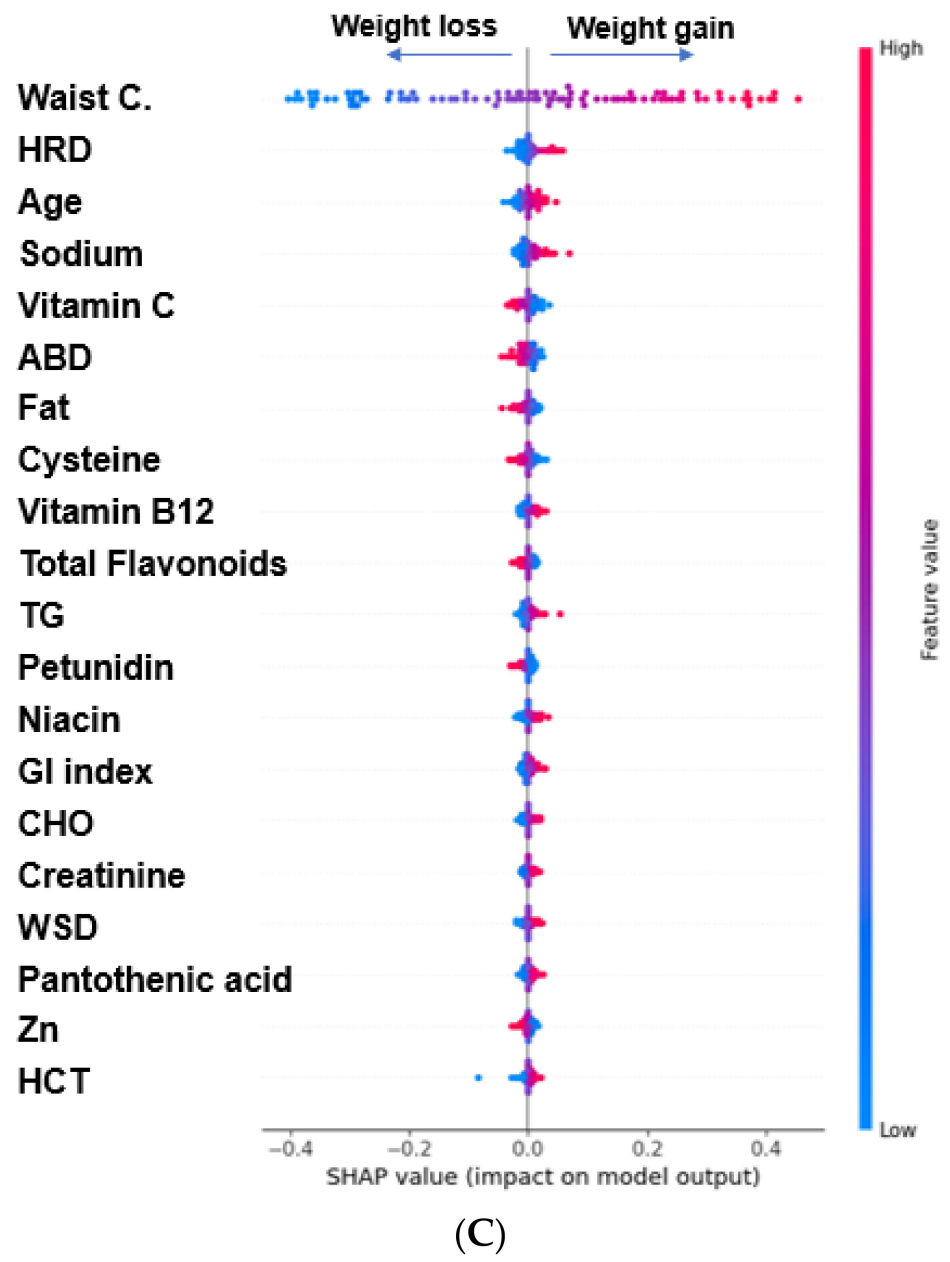
The relative importance of the top 20 variables for predicting the waist circumference (WC) reduction. (**A**) WC reduction model generated by the XGBoost algorithm; (**B**) WC reduction model generated by the random forest algorithm; (**C**) WC reduction model generated by the deep neural network (DNN). Grip force: mean of grip strength in the right and left hands; C. circumference; BUN, blood urinary nitrogen; SBP, systolic blood pressure, WBC, white blood cell count. ABD, Asian-style balanced diet; HDL, high-density lipoprotein; TG, triglyceride; HRD, diet with high rice consumption; Hs-CRP, high-sensitivity C-reactive protein; γ-GTP, γ-glutamyl transpeptidase; U., urinary; HB, hemoglobin; HCT, hematocrit; PSA, prostate-specific antigen; GI, glycemic; CHO, carbohydrate; WSD, Western-style diet.

The 223 parameters were manually chosen to explore the prediction model for WC reduction. The primary biomarkers were searched using three ML algorithms. Out of the participant group, 10% (n = 6561) were separated for the validation set before starting ML. The remaining participants were used to explore the biomarkers that influence reductions in WC. They were divided into training (*n* = 47,240) and test sets (*n* = 11,810) at an 8:2 ratio. The AUROC was 0.793–0.866 in the logistic regression, XGboost, random forest, and DNN algorithms (Table 4). The prediction models included 20 variables to explain the reductions in WC. The models included some of the same variables but also some different variables generated by the XGboost, random forest, and DNN algorithms. The variables in the prediction model selected by the XGBoost algorithm were WC, gender, hip circumference, grip force, pulse, weight, age, dietary patterns including the ABD and HRD, serum γ-GTP, BUN, TG, HDL-C, LDL-C, serum fibrinogen, platelet, hs-CRP, creatinine concentration, and pulse rate (Figure 5A). Those selected by the random forest algorithm included WC, weight, hip circumferences, gender, grip force, height, serum γ-GTP, HDL-C, TG, hs-CRP, alanine aminotransferase (ALT), creatinine, HbA1c, glucose concentration, SBP, urinary uric acid concentration, smoking status, and alcohol drinking status (Figure 5B). However, the random forest algorithm did not include dietary intake or dietary patterns. In the DNN procedure, WC, age, gender, hip circumference, serum TG and creatinine concentration, hematocrit, HRD, ABD, WSD, glycemic index, vitamin C, pantothenic acid, sodium, carbohydrates, fats, total flavonoids, petunidin, cyanidin, zinc, and cysteine were included (Figure 5C). Even the same variables showed different relative scores in different prediction models. The AUROC, accuracy, and k-fold were higher in the prediction model generated with the XGBoost algorithm than in the other models. Therefore, the XGBoost model was a more reliable predictor of WC reduction.

## 4. Discussion

The present study examined the longitudinal relationship between WC and dietary factors and yielded insightful findings. The inverse correlation observed between changes in WC and diet quality, as measured by the MHEI, highlights the pivotal role of a healthy diet in achieving sustainable reductions in WC. Furthermore, our study identified the specific dietary patterns influencing WC dynamics over an approximately 5-year period. Notably, the positive associations of ABD with decreased WC, alongside WC’s inverse relationships with the WSD and HRD, emphasize the nuanced impact of dietary choices. The merits of our research lie in its longitudinal design, which provides a comprehensive understanding of the effects of a sustainable and healthy diet as measured by a SQFFQ. By quantifying food intake over the last year, our study introduced a temporal dimension, underscoring the importance of sustained dietary habits in outcomes associated with changes in WC. Therefore, our findings highlighted the significance of diet quality and specific dietary patterns in preventing and managing abdominal obesity, thus offering valuable insights for tailored interventions and long-term strategies.

Abdominal obesity is a well-known risk factor for metabolic disease [24]. Strategies that aid the sustained reduction and maintenance of abdominal obesity assessed by WC can prevent or delay the progression of MetS and associated chronic diseases. Long-term studies have shown that a very low-calorie-restricted diet does not continuously improve abdominal obesity since it decreases not only visceral fat mass [25] but also muscle mass. In addition, it is a challenge to sustain such low-calorie diets over an extended period [26]. Studies using different dietary interventions, including increases in *w*–3 polyunsaturated fatty acids (PUFA) but not protein intake, have been conducted to decrease visceral fat mass and improve abdominal obesity [27,28]. Intermittent fasting and ketogenic diets have also been considered to reduce abdominal obesity [29,30]. However, the potential risk and long-term effectiveness of these interventions remain unknown [26]. Dietary quality is pivotal in reducing WC in interventions involving energy restrictions. In one study, a high-quality diet showed a more significant reduction in WC than a low-quality diet during a 12-week intervention with a 25% energy restriction [31]. The present study demonstrated that a high MHEI score was positively associated with reductions in WC. However, energy intake was not significantly different between the WC-loss (85.5% of the estimated energy requirement for men and 93.5% for women) and WC-gain groups (84.8% for men and 94.0% for women). The results of the Korea National Health and Nutrition Examination Survey (KNHANES) 2013–2016 on the association between the MHEI score and the risk of abdominal obesity were similar to our current study [6]. Among the MHEI score components, adequacy and moderation scores, but not balance scores, were positively linked to reductions in WC. Therefore, a high-quality diet is critical for a sustainable decrease in abdominal obesity.

For many people, adopting a high-quality diet can be challenging. It becomes imperative to explore dietary patterns that are not only more feasible but also comprehensible in real-world scenarios [32]. Our study extended beyond investigating the positive correlation between a high-quality diet and a reduction in WC. It explored alternative dietary patterns, recognizing the crucial role of practicality in adopting such diets. However, in exploring the biomarkers associated with reductions in WC using the ML approach, high MHEI score was not included in the prediction models. However, dietary patterns, including ABD and WSD, were included. This suggested that dietary patterns could be practically applied for a sustainable reduction in WC. This is essential, as its feasibility is a crucial determinant of adherence and sustainability [33]. Our study categorized participants into four dietary patterns, indicating significant associations with WC changes. Specifically, participants adhering more to the ABD exhibited a greater decrease in WC than those following the ABD less. However, those adhering to the WSD and HRD exhibited an opposing trend. The PBD also exhibited a trend toward WC reduction without reaching statistical significance. The ABD has a high MHEI score in the KNHANES 2013–2016 [6]. Consistent with the cross-sectional study results, the ABD significantly reduced obesity, abdominal obesity, and dyslipidemia in overweight and obese Korean adults, compared to an American balanced diet in a randomized clinical trial comparing the ABD with the balanced American diet [9,34].

Insights from studies on the use of well-established dietary patterns, such as the Mediterranean and DASH diets [4,32], have shown promise in achieving sustainable reductions in WC. However, their applicability to Asian diets, including Korean ones, poses challenges. As we recognize the diversity in dietary patterns across countries, our research emphasizes that each nation may have a unique, sustainable dietary approach to reducing WC. The PBD, potentially close to the Mediterranean diet, exhibited no significant reductions in WC. In contrast, the ABD resulted in practical and effective reductions in WC in Koreans in the present study. ABD was close to the DASH diet, including grain, beans, vegetables, fermented vegetables (kimchi), beans, mushrooms, fish, seafood, and lean meats. However, salt intake (6 g/day) was not as restricted here as with DASH (2.3 g/day). By incorporating diverse dietary patterns and considering their practicality in real-world scenarios, our results demonstrated an understanding of effective strategies for abdominal obesity management, offering valuable insights for both researchers and individuals seeking sustainable approaches to dietary modification.

The MHEI was modified to include items reflecting the ABD and has been linked to improving abdominal obesity and MetS in previous studies [6,14,35]. The ABD contains a high concentration of kimchi, vegetables, mushrooms, fish, crab, meat, seaweed, and pickles. Despite the documented benefits of ABD, the disadvantages of the high salt content in the soups and fermented foods that are part of ABD have been underscored. High salt intake has been positively associated with the risk of MetS in the KNHANES and KoGES [6,36]. However, previous studies have also revealed that the ABD is inversely associated with MetS risk. However, strong adherence to the ABD would result in a higher salt consumption than weak adherence to the ABD [6]. Furthermore, a high intake of fermented bean products is reported to be inversely associated with the risk of abdominal obesity [5,37,38], suggesting that these products could alleviate the harmful effect of sodium. However, the present study showed that sodium intake did not differ between the WC-loss and WC-gain groups. However, the biomarkers for WC reductions identified by the ML approach included sodium intake, which was inversely associated with a reduction in WC. These results might be linked to the sodium reduction strategy implemented in Korean meals since 2016, enforced after the follow-up study data were collected between 2012 and 2016. Therefore, the decreased sodium intake in the ABD promoted reductions in the WC.

The genetic variants associated with obesity and abdominal obesity were identified among participants of the city-hospital-based cohort during the initial recruitment [22]. Although two sets of PRSs were derived from the genetic variants linked to abdominal obesity, they were not selected in the prediction models for WC reductions generated using the ML approach [22]. Interestingly, no significant differences in reductions in WC were exhibited among individuals with different PRSs for abdominal obesity-related genetic variant set 1 (AO1_PRS). However, individuals with a high PRS for the AO2_PRS set experienced a notable reduction in WC compared to those with a low PRS. This suggests that the genetic variants in the AO2_PRS set are more related to general obesity, while the genetic variants selected in set 1 with adjustments for LBM are specifically associated with abdominal obesity. Individuals with abdominal obesity tended to demonstrate a reduction in WC during the first recruitment stage. The PRSs of the AO2_PRS set did not hinder this reduction, while the PRSs of the AO1_PRS set exhibited some inhibitory effect on WC reduction. Therefore, overall, genetics did not appear to exert a substantial influence on abdominal obesity, and the ability to reduce WC by lifestyle modification can potentially override these genetic factors.

This study has several strengths. First, this study was conducted as part of a longitudinal investigation spanning about 5 years, providing a comprehensive understanding of how sustained reductions in abdominal obesity can be achieved. The incorporation of a large city-hospital-based cohort enhances the generalizability of the findings. We employed ML techniques to develop predictive models for reducing WC, allowing for the integration of diverse factors. Furthermore, our exploration achieved depth by including genetic, biochemical, and environmental variables and identifying novel biomarkers. The positive association observed between a high-quality diet, particularly the ABD, and decreases in WC underscores the practical implications of our study for developing preventive and management strategies. Overall, the strengths of the study confirm the reliability and significance of our findings for understanding the factors that influence abdominal obesity and developing effective interventions. However, the study has some limitations. First, the reliance on self-reported dietary data introduces the possibility of recall bias, as it may inaccurately capture the full spectrum of participants’ food intake. However, a skilled technician determined the food intake using well-validated tools. Second, the composition of the study cohort, predominantly comprising individuals from a city hospital setting, may impact the generalizability of our findings to broader populations with differing demographic characteristics or health profiles. Finally, while the PRSs utilized may not encapsulate the entirety of the genetic factors contributing to abdominal obesity, it is noteworthy that the observed genetic impact appeared to be minimal in the participants. These limitations should be considered when interpreting the results and designing future studies to understand better the factors influencing abdominal obesity.

## 5. Conclusions

The present study offered valuable insights into the complex interplay of genetic, environmental, and lifestyle factors influencing changes in abdominal obesity. We developed predictive models using ML techniques using a longitudinal approach for about 5 years and incorporating a diverse set of 220 variables. Notably, the impactful role of a high-quality diet, as indicated by a high MHEI, reduced abdominal obesity. Sustained adherence to the ABD, characterized by a high MHEI, emerged as a practical and effective strategy for managing and preventing abdominal obesity. These findings highlight the significance of adopting and maintaining a high-quality diet with a high MHEI as an actionable approach to curb the prevalence of abdominal obesity.

## Figures and Tables

**Table 1:** Anthropometry and biochemical parameters according to waist circumference (WC) change and gender at the second recruitment stage.

	Men (*n* = 22,290)		Women (*n* = 43,321)	
	WC-Loss (*n* = 11,670)	WC-Gain (*n* = 10,620)	WC-Loss (*n* = 20,527)	WC-Gain (*n* = 22,794)
Age (years)	61.8 ± 0.08 ^a^	61.2 ± 0.08 ^b^	57.4 ± 0.06 ^c^	57.1 ± 0.05 ^d^***^+++#^
Gender [N (%)] ^1^	11,670 (36.3) ^1^	10,620 (31.8)	20,527 (63.8)	22,794 (68.2) ^+++^
Duration (years)	4.87 ± 0.12	4.82 ± 0.23	4.87 ± 0.22	4.80 ± 0.21
Education [N (%)] ^1^				
≤Middle school	2754 (23.6)	2007 (18.9)	4559 (22.2)	3899 (17.1)
High school	8182 (70.1)	7880 (74.2)	14,584 (71.0)	17,189 (75.4)
≥Collage	710 (6.36)	728 (6.86) ^+++^	1383 (6.73)	1706 (7.48) ^++^
Waist C changes (cm)	−4.0 ± 0.04 ^d^	4.18 ± 0.04 ^b^	−4.32 ± 0.03 ^c^	5.08 ± 0.03 ^a^***^+++###^
Height (cm)	168.6 ± 0.06 ^a^	168.5 ± 0.06 ^a^	156.5 ± 0.04 ^b^	156.5 ± 0.04 ^b^***
Waist C (cm)	82.5 ± 0.08 ^b^	87.2 ± 0.09 ^a^	76.6 ± 0.06 ^d^	81.8 ± 0.05 ^c^***^+++###^
Hip C (cm)	94 ± 0.06 ^b^	96.2 ± 0.06 ^a^	91.7 ± 0.04 ^c^	93.9 ± 0.04 ^b^***^+++^
BMI (kg/m^2^)	23.9 ± 0.03 ^c^	24.5 ± 0.03 ^a^	23.4 ± 0.02 ^d^	24 ± 0.02 ^b^***^+++^
Hemoglobin (g/dL)	14.95 ± 0.01 ^d^	15.08 ± 0.01 ^c^	13.32 ± 0.01 ^b^	13.35 ± 0.01 ^a^***^+++###^
Hematocrit (%)	44.3 ± 0.03 ^b^	44.6 ± 0.03 ^a^	40.2 ± 0.02 ^d^	40.3 ± 0.02 ^c^***^+++###^
MetS [N (%)] ^1^	8770 (45.7)	1850 (59.7) ^+++^	19,931 (51.4)	2863 (63.2) ^+++^
Glucose (mg/dL)	103 ± 0.21 ^a^	102.7 ± 0.22 ^a^	99 ± 0.15 ^b^	99.1 ± 0.14 ^b^***
HbA1c (%)	5.64 ± 0.01 ^b^	5.64 ± 0.01 ^b^	5.69 ± 0.01 ^a^	5.69 ± 0.01 ^a^***
Total cholesterol (mg/dL)	189 ± 0.4 ^d^	192 ± 0.42 ^c^	203 ± 0.28 ^b^	205 ± 0.27 ^a^***^+++^
HDL (mg/dL)	53.8 ± 0.16 ^c^	52.5 ± 0.17 ^d^	61 ± 0.11 ^a^	60.1 ± 0.11 ^b^***^+++^
LDL (mg/dL)	111 ± 0.37 ^d^	113 ± 0.38 ^c^	118 ± 0.26 ^b^	120 ± 0.25 ^a^***^+++^
Triglyceride (mg/dL)	122 ± 0.77 ^b^	132 ± 0.81 ^a^	120 ± 0.54 ^c^	124 ± 0.52 ^b^***^+++###^
SBP (mmHg)	124 ± 0.16 ^b^	126 ± 0.16 ^a^	121 ± 0.11 ^d^	123 ± 0.11 ^c^***^+++##^
DBP (mmHg)	75.9 ± 0.1 ^b^	77.1 ± 0.11 ^a^	73.3 ± 0.07 ^d^	74.1 ± 0.07 ^c^***^+++^
Creatinine	0.97 ± 0.003 ^a^	0.98 ± 0.003 ^a^	0.7 ± 0.002 ^b^	0.7 ± 0.002 ^b^***^++^
BUN (mg/dL)	16 ± 0.05 ^a^	15.9 ± 0.05 ^a^	14.7 ± 0.03 ^b^	14.7 ± 0.03 ^b^***
eGFR (mL/min/1.73 m^2^)	82.8 ± 0.17 ^c^	82.3 ± 0.18 ^c^	88.9 ± 0.12 ^a^	88.5 ± 0.12 ^b^***^++^
γ-GTP (mg/dL)	35.4 ± 0.42 ^b^	37.1 ± 0.44 ^a^	26.3 ± 0.3 ^d^	27.4 ± 0.29 ^c^***^+++^
Fibrinogen (mg/dL)	305 ± 0.65 ^b^	302.7 ± 0.67 ^c^	327.2 ± 0.46 ^a^	326.3 ± 0.44 ^a^***^++^
Platelet (Thous/uL)	232.1 ± 0.64 ^a^	232.4 ± 0.67 ^a^	259.6 ± 0.45 ^c^	262.9 ± 0.43 b
Hs-CRP (ng/mL)	0.13 ± 0.004 ^a^	0.117 ± 0.004 ^b^	0.115 ± 0.003 ^c^	0.114 ± 0.003 ^c^*^+#^
Grip force (N)	35.4 ± 0.07 ^a^	35.7 ± 0.07 ^a^	21.3 ± 0.05 ^b^	21.3 ± 0.04 ^b^***^#^
MI [N (%)] ^1^	263 (2.25) ^1^	209 (1.97)	194 (0.95)	244 (1.07)
Stroke [N (%)] ^1^	96 (0.81) ^1^	73 (0.69)	74 (0.36)	75 (0.33)
CVD [N (%)] ^1^	619 (5.33) ^1^	504 (4.75) ^+^	542 (2.65)	507 (2.23) ^++^

Values represent adjusted means ± standard errors, or the number of participants (their percentages) ^1^. Adjusted by age, residence, region, education, income, follow-up duration, drinking status, smoking status, and exercise. WC-loss, <0 change in WC between the second and the first measurements; WC-gain, ≥0 change in WC between the second and the first measurements. C, circumference; BMI, body mass index; MetS, metabolic syndrome; γ-GTP, γ-glutamyl transpeptidase; BUN, blood urinary nitrogen; eGFR, estimated glomerular filtration rate; Hs-CRP, high-sensitivity C-reactive protein; MI, myocardial infarction; CVD, cardiovascular disease. * Significant differences by gender at *p* < 0.05 and *** at *p* < 0.001. ^+^ Significant differences by the WC change at *p* < 0.05, ^++^ at *p* < 0.01 and ^+++^ at *p* < 0.001. ^#^ Significant interaction between gender and WC change at *p* < 0.05 and ^###^ at *p* < 0.001. ^#^ Significant interaction between gender and WC change at *p* < 0.05, ^##^ at *p* < 0.01, ^###^ at *p* < 0.001. ^a,b,c,d^ Different letters indicate significant differences among the groups in Tukey’s test results at *p* < 0.05.

**Table 2:** Nutrient intake according to waist circumference (WC) change and gender at the follow-up study, adjusted by age, residence region, education, income, follow-up duration, energy intake, drinking status, smoking status, and exercise at the second recruitment stage.

	Men (*n* = 22,290)		Women (*n* = 43,321)	
	WC-Loss (*n* = 11,670)	WC-Gain (*n* = 10,620)	WC-Loss (*n* = 20,527)	WC-Gain (*n* = 22,794)
Energy intake (EER %)	85.5 ± 0.3 ^b^	84.8 ± 0.32 ^b^	93.5 ± 0.21 ^a^	94 ± 0.2 ^a^***^##^
Carbohydrate (En%)	71.4 ± 0.08 ^a^	71.3 ± 0.08 ^a^	71 ± 0.05 ^b^	70.9 ± 0.05 ^b^***
Fat (En%)	14.3 ± 0.06 ^b^	14.4 ± 0.06 ^b^	14.7 ± 0.04 ^a^	14.8 ± 0.04 ^a^***^+^
SFA (En%)	8.04 ± 0.06 ^a^	8.06 ± 0.06 ^a^	7.39 ± 0.04 ^b^	7.44 ± 0.04 ^b^***
MUFA (En%)	10.3 ± 0.07 ^a^	10.3 ± 0.08 ^a^	9.16 ± 0.05 ^b^	9.3 ± 0.05 ^b^***
PUFA (En%)	4.87 ± 0.03 ^a^	4.89 ± 0.03 ^a^	4.33 ± 0.02 ^b^	4.38 ± 0.02 ^b^***
Protein (%)	13.2 ± 0.03 ^b^	13.1 ± 0.03 ^b^	13.5 ± 0.02 ^a^	13.5 ± 0.02 ^a^***^+^
Fiber (mg/day)	11.6 ± 0.05 ^b^	11.6 ± 0.05 ^b^	12.3 ± 0.03 ^a^	12.2 ± 0.03 ^a^***
Calcium (mg/day)	353 ± 1.82 ^b^	352 ± 1.91 ^b^	421 ± 1.28 ^a^	420 ± 1.23 ^a^***
Sodium (g/day)	1.92 ± 0.01 ^b^	1.93 ± 0.01 ^b^	1.97 ± 0.01 ^a^	1.96 ± 0.01 ^a^***
Potassium (g/day)	1.88 ± 0.01 ^b^	1.87 ± 0.01 ^b^	2.08 ± 0.01 ^a^	2.08 ± 0.01 ^a^***
Zinc (mg/day)	7.20 ± 0.02 ^c^	7.12 ± 0.02 ^d^	7.53 ± 0.02 ^a^	7.40 ± 0.01 ^b^***^+++^
Vitamin C (mg/day)	84.3 ± 0.52 ^c^	81.8 ± 0.54 ^d^	98.9 ± 0.37 ^a^	97.2 ± 0.35 ^b^***^+++^
Vitamin B6 (mg/day)	1.37 ± 0.004 ^b^	1.36 ± 0.004 ^b^	1.47 ± 0.002 ^a^	1.46 ± 0.002 ^a^***^++^
Niacin (mg/day)	13.2 ± 0.03 ^b^	13.1 ± 0.03 ^b^	13.6 ± 0.02 ^a^	13.5 ± 0.02 ^a^***
Folate (ug/day)	177 ± 0.82 ^c^	175 ± 0.85 ^d^	199 ± 0.58 ^a^	197 ± 0.56 ^b^***^+++^
Vitamin B12 (ug/day)	6.87 ± 0.05 ^b^	6.91 ± 0.05 ^b^	7.89 ± 0.04 ^a^	7.99 ± 0.04 ^a^***
Pantothenic acid (mg/day)	2.54 ± 0.01 ^b^	2.53 ± 0.01 ^b^	2.92 ± 0.01 ^a^	2.90 ± 0.01 ^a^***
Vitamin A (RE ug/day)	396 ± 2.54 ^c^	390 ± 2.66 ^c^	445 ± 1.80 ^a^	438 ± 1.73 ^b^***^++^
Vitamin D (ug/day)	29.4 ± 0.27 ^c^	28.8 ± 0.28 ^c^	36.8 ± 0.19 ^a^	36 ± 0.18 ^b^***^++^
Vitamin K (ug/day)	53.3 ± 0.62 ^b^	51.4 ± 0.65 ^c^	61.1 ± 0.44 ^a^	60.3 ± 0.42 ^a^***^++^
DII	−14.3 ± 0.94 ^a^	−14.5 ± 0.82 ^a^	−18.6 ± 0.7 ^b^	−18 ± 0.62 ^b^***
Total flavonoids (ug/day)	35.4 ± 0.28 ^b^	34.2 ± 0.29 ^c^	43.1 ± 0.19 ^a^	43 ± 0.19 ^a^***^++#^
Glycemic index	49.2 ± 0.1 ^b^	49.8 ± 0.1 ^a^	46.3 ± 0.07 ^d^	46.8 ± 0.07 ^c^***^+++^
ABD [N (%)] ^1^	6267 (48.5)	4059 (46.9) ^+^	15215 (53.3)	6480 (51.4) ^+++^
WSD [N (%)] ^1^	5231 (45.4)	5095 (50.6) ^+++^	15537 (52.2)	6158 (54.2) ^+++^
PBD [N (%)] ^1^	8293 (48.2)	2033 (46.3) ^+^	12936 (52.6)	8759 (52.9)
HRD [N (%)] ^1^	6753 (46.2)	3573 (50.7) ^+++^	13998 (52.0)	7697 (54.1) ^+++^
Coffee (g/day)	3.95 ± 0.04 ^b^	4.09 ± 0.04 ^a^	3.65 ± 0.03 ^d^	3.84 ± 0.03 ^c^***^+++^
Alcohol (g/week)	2.33 ± 0.03 ^b^	2.65 ± 0.03 ^a^	0.58 ± 0.02 ^c^	0.61 ± 0.02 ^c^***^+++###^
Exercise [N (%)] ^1^	7259 (62.2)	6328 (59.6) ^+++^	12,052 (58.7)	12,780 (56.1) ^+++^
Former smoking	4994 (44.0)	4313 (41.4)	195 (0.97)	258 (1.16)
Smoking [N (%)] ^1^	2788 (24.6)	2870 (27.6) ^+++^	324 (1.61)	384 (1.72)

Values represent adjusted means ± standard errors or the number of participants (percentage) ^1^. Adjusted by the covariates such as age, residence, region, education, income, follow-up duration, drinking status, smoking status, and exercise. WC-loss, change <0 in WC between the second and the first measurements; WC-gain, ≥0 change in WC between the second and the first measurements. EER, estimated energy requirement; En%, energy percentage; SFA, saturated fatty acids; MUFA, monounsaturated fatty acids; PUFA, polyunsaturated fatty acids; DII, dietary inflammatory index; ABD, Asian-style balanced diet; PBD, plant-based diet; WSD, Western-style diet; HRD, diet with high rice consumption. *** Significant differences by gender at *p* < 0.001. ^+^ Significant differences by the WC change at *p* < 0.05, ^++^ at *p* < 0.01, and ^+++^ at *p* < 0.001. ^#^ Significant interaction between gender and WC change at *p* < 0.05, ^##^ at *p* < 0.01, and ^###^ at *p* < 0.001. ^a,b,c,d^ Different letters indicate significant differences among the groups in Tukey’s test results at *p* < 0.05.

**Table 3:** Adjusted means and 95% CI for the modified Healthy Eating Index (MHEI) scores at the second recruitment stage according to gender and waist circumference (WC) reduction.

Classification	Men (*n* = 22,290)		Women (*n* = 43,321)		*p* Value for WC Changes *	*p* Value for Gender ^+^
	WC-Loss (*n* = 11,670)	WC-Gain (*n* = 10,620)	WC-Loss (*n* = 20,527)	WC-Gain (*n* = 22,794)		
Having breakfast	9.18 (9.11–9.24) ^a^	9.10 (9.03–9.16) ^b^	8.63 (8.59–8.68) ^c^	8.56 (8.52–8.50) ^c^	0.0025	<0.0001
Mixed grains intake	4.14 (4.11–4.18) ^b^	4.07 (4.03–4.11) ^c^	4.43 (4.41–4.46) ^a^	4.41 (4.39–4.44) ^a^	0.0022	<0.0001
Total fruit intake	2.81 (2.77–2.84) ^c^	2.62 (2.58–2.66) ^d^	3.76 (3.74–3.79) ^a^	3.71 (3.69–3.74) ^b^	<0.0001	<0.0001
Vegetable intake, excluding kimchi and pickled vegetables	2.57 (2.55–2.59) ^b^	2.48 (2.46–2.50) ^c^	3.07 (3.06–3.08) ^a^	3.07 (3.06–3.08) ^a^	<0.0001	<0.0001
Fermented vegetable intake	3.57 (3.53–3.61) ^a^	3.52 (3.48–3.57) ^a^	3.38 (3.35–3.41) ^b^	3.32 (3.30–3.35) ^c^	0.0013	<0.0001
Seaweed intake	2.39 (2.35–2.43) ^b^	2.33 (2.28–2.37) ^b^	2.88 (2.86–2.91) ^a^	2.88 (2.85–2.90) ^a^	0.0348	<0.0001
Fish intake	0.744 (0.708–0.779) ^c^	0.707 (0.669–0.744) ^c^	1.613 (1.588–1.638) ^a^	1.530 (1.506–1.555) ^b^	<0.0001	<0.0001
Meat and eggs	2.94 (2.91–2.98) ^b^	2.94 (2.90–2.97) ^b^	3.49 (3.47–3.52) ^a^	3.50 (3.48–3.52) ^a^	0.8825	<0.0001
Beans, including fermented beans	1.01 (0.97–1.05) ^b^	1.02 (0.98–1.07) ^b^	2.13 (2.11–2.16) ^a^	2.13 (2.11–2.16) ^a^	0.6649	<0.0001
Milk and milk products	1.33 (1.29–1.38) ^b^	1.36 (1.31–1.40) ^b^	1.94 (1.91–1.97) ^a^	1.95 (1.92–1.98) ^a^	0.2577	<0.0001
Nuts	2.28 (2.25–2.31) ^b^	2.25 (2.22–2.28) ^b^	2.39 (2.37–2.41) ^a^	2.39 (2.38–2.41) ^a^	0.1686	<0.0001
Total MHEI for adequacy	30.8 (30.7–31.0) ^c^	30.3 (30.2–30.5) ^d^	35.2 (35.1–35.3) ^a^	35.0 (34.9–35.1) ^b^	<0.0001	<0.0001
Saturated fatty acids (En%)	8.69 (8.63–8.75)	8.73 (8.66–8.79)	8.76 (8.71–8.80)	8.78 (8.74–8.82)	0.1890	0.0633
Polyunsaturated fatty acids (En%)	4.31 (4.28–4.33)	4.29 (4.27–4.32)	4.26 (4.24–4.28)	4.26 (4.24–4.27)	0.3638	0.0047
Sodium intake	8.29 (8.24–8.34) ^a^	8.26 (8.21–8.31) ^a^	8.21 (8.17–8.24) ^b^	8.19 (8.16–8.22) ^b^	0.2584	0.0023
Sweets and beverages (En%)	6.16 (6.12–6.19) ^a^	6.12 (6.09–6.16) ^a^	5.97 (5.95–6.00) ^b^	5.96 (5.93–5.98) ^b^	0.0906	<0.0001
Fast foods (En%)	3.67 (3.63–3.71) ^a^	3.54 (3.50–3.58) ^b^	3.63 (3.60–3.66) ^a^	3.56 (3.54–3.59) ^b^	<0.0001	0.6953
Noodles (En%)	2.72 (2.69–2.76) ^c^	2.64 (2.60–2.68) ^d^	3.01 (2.99–3.04) ^a^	2.94 (2.92–2.97) ^b^	<0.0001	<0.0001
Total MHEI for moderation	45.2 (45.1–45.3) ^a^	44.9 (44.8–45.1) ^b^	45.0 (44.9–45.1) ^a^	44.9 (44.8–45.0) ^b^	<0.0001	0.1369
Energy intake	3.65 (3.61–3.69) ^a^	3.58 (3.54–3.63) ^a^	3.49 (3.45–3.52) ^b^	3.49 (3.46–3.52) ^b^	0.0579	<0.001
Vitamin C intake	2.05 (2.01–2.10) ^b^	1.96 (1.91–2.00) ^c^	2.55 (2.52–2.58) ^a^	2.51 (2.48–2.54) ^a^	<0.0001	<0.0001
Fiber intake	0.56 (0.53–0.59) ^c^	0.58 (0.55–0.61) ^c^	1.99 (1.97–2.01) ^a^	1.95 (1.93–1.97) ^b^	0.4450	<0.0001
Calcium intake	0.196 (0.170–0.223) ^b^	0.211 (0.183–0.239) ^b^	0.762 (0.743–0.781) ^a^	0.770 (0.752–0.788) ^a^	0.2808	<0.0001
Carbohydrates (En%)	1.32 (1.28–1.36) ^b^	1.30 (1.25–1.34) ^b^	1.55 (1.52–1.58) ^a^	1.54 (1.51–1.57) ^a^	0.3171	<0.0001
Fat (En%)	3.06 (3.02–3.10) ^b^	3.09 (3.05–3.13) ^b^	3.23 (3.21–3.26) ^a^	3.26 (3.24–3.29) ^a^	0.0814	<0.0001
Total MHEI for balance	10.8 (10.7–10.9) ^b^	10.7 (10.5–10.8) ^b^	13.6 (13.6–13.7) ^a^	13.6 (13.5–13.7) ^a^	0.0586	<0.0001
Total MHEI	86.8 (86.6–87.1) ^c^	85.9 (85.6–86.1) ^d^	93.9 (93.7–94.0) ^a^	93.5 (93.3–93.6) ^b^	<0.0001	<0.0001

WC-loss, <0 change in WC between the second and the first measurements; WC-gain, ≥0 change in WC between the second and the first measurements. Adjusted by age, residence, region, education, income, follow-up duration, drinking status, smoking status, and exercise. Fermented beans included chungkookjang, doenjang, kochujang, and soy sauce, and fermented vegetables included kimchi and pickles. En%, energy percent. *^,+^ Two-way ANOVA with covariates according to WC change (*) and gender (^+^). ^a,b,c,d^ Different letters indicate significant differences among the groups in Tukey’s test results at *p* < 0.05. CI, confidence intervals; total fruits: fresh fruits and fruit juice.

**Table 4:** The area under the receiver operating characteristic curve (AUROC), accuracy, and k-fold of prediction models containing 20 features for waist circumference (WC) loss using machine learning algorithms at the second recruitment stage.

	Logistic Regression	XGBoost	Random Forest	DNN
AUROC	0.793 (0.792–0.794)	0.866 (0.864–0.867)	0.795 (0.794–0.795)	0.811
Accuracy	0.721 (0.721–0.722)	0.828 (0.827–0.828)	0.735 (0.735–0.736)	0.805
k-fold	0.721 (0.717–0.725)	0.845 (0.831–0.860)	0.766 (0.763–0.770)	0.78

To evaluate model performance, we allocated 10% of the data for a validation set, and then the remaining 90% was utilized for training and testing at an 8:2 ratio. The dataset was further standardized, resulting in 220 variables, and training and test sets were generated using the randomized grid search method. The prediction model with the top 20 variables was generated from logistic regression, extreme gradient boosting (XGBoost), random forest, and deep neural network (DNN) for WC reduction.

## Data Availability

The raw data involved in this study will be available in Korea Biobank at Osong, Korea.

## Supplementary Materials

The following supporting information can be downloaded at: https://www.mdpi.com/article/10.3390/nu16040534/s1, Table S1: Criteria of modified Health Eating Index (MHEI); Figure S1: Body mass index (BMI) scores and waist circumferences (WC) at the first recruitment stage according to gender and WC change.
