# Supplementary material for: Association of a High Healthy Eating Index Diet with Long-Term Visceral Fat Loss in a Large Longitudinal Study
Source: Nutrients. 2024 Feb 14;16(4):534. doi: 10.3390/nu16040534 (PMC10892686; doi:10.3390/nu16040534)
Supplement: Supplementary file 1 [file nutrients-16-00534-s001.zip › nutrients-2875053-supplementary.pdf]

Table S1. Criteria of Korea Health Eating Index (KHEI)

Components(score range)	Standard maximum score	score
Adequacy		
Have breakfast	5-7 times/w	10
	3- <5 times/w	7
	1- <3 times/w	4
	0 times	0
Mixed grains intake	≥0.8 serving/d	5
	0.1-<0.8 serving/d	3
	<0.1	0
Fresh fruit intake	• Men aged 19-64 years: ≥ 1.5 serving/d • Women aged 19-64 years: ≥ 1 serving/d • Men aged 65 years and overs: ≥ 1 serving/d • Women aged 65 years and over: ≥ 0.5 serving/d	5
	• Men aged 19-64 years: 0.5-1.5 serving/d • Women aged 19-64 years: 0.3-1 serving/d • Men aged 65 years and overs: 0.3- 1 serving/d • Women aged 65 years and over: 0.2-0.5 serving/d	3
	• Men aged 19-64 years: 0-0.5 serving/d • Women aged 19-64 years: 0-0.3 serving/d • Men aged 65 years and overs: 0.3-1 serving/d • Women aged 65 years and over: 0.2-0.5 serving/d	0
Vegetable intake excluding Kimchi and pickled vegetables intake	• Men and women aged 19-64 years: ≥ 5 serving/d • Men aged 65 years and overs: ≥ 5 serving/d • Women aged 65 years and overs: ≥ 3 serving/d	5
	• Men and women aged 19-64 years: 2-< 5 serving/d • Men aged 65 years and overs: 2-< 5 serving/d • Women aged 65 years and overs: 2-< 3 serving/d	3
	• Men and women aged 19-64 years: <2 serving/d • Men aged 65 years and overs: <2 serving/d • Women aged 65 years and overs: <2 serving/d	0
Fermented vegetables kimchi and pickled vegetable	≥3 serving/d	5
	1-<3 serving/d	3
	<1 serving/d	0
Seaweed intake	≥ 2.5 serving/week	5
	0.5-2.4 serving/week	3
	<0.5 serving/week	0
Fish	• Men aged 19-64 years: ≥ 2 serving/d • Women aged 19-64 years: ≥ 1.5 serving/d • Men aged 65 years and overs: ≥ 1.5 serving/d • Women aged 65 years and overs: ≥ 1 serving/d	5
	• Men aged 19-64 years: 1-2 serving/d • Women aged 19-64 years: 1-2 serving/d • Men aged 65 years and overs: 1-1.5 serving/d • Women aged 65 years and overs: 0.5-1 serving/d	3
	• Men aged 19-64 years: <1 serving/d • Women aged 19-64 years: <1 serving/d • Men aged 65 years and overs: <1 serving/d • Women aged 65 years and overs: <0.5 serving/d	0
Meat & eggs	• Men aged 19-64 years: 2-<3 serving/d • Women aged 19-64 years: 1.5-<2 serving/d • Men aged 65 years and overs: 1.5-<2 serving/d •Women aged 65 years and overs: 1.5-2 serving/d	5
	• Men aged 19-64 years: <2 serving/d • Women aged 19-64 years: <1.5 serving/d • Men aged 65 years and overs: <1.5 serving/d • Women aged 65 years and overs: < 1.5 serving/d	3
	• Men aged 19-64 years: ≥4 serving/d • Women aged 19-64 years: ≥ 3 serving/d • Men aged 65 years and overs: ≥ 3 serving/d • Women aged 65 years and overs: ≥ 2.5 serving/d	0
Beans including	• Men aged 19-64 years: ≥ 1 serving/d • Women aged 19-64 years: ≥ 1	5

fermented beans	serving/d • Men aged 65 years and overs: ≥ 0.5 serving/d • Women aged 65 years and overs: ≥ 0.5 serving/d	
	• Men aged 19-64 years: 0.5-<1 serving/d • Women aged 19-64 years: 0.5-<1 serving/d • Men aged 65 years and overs: <0.5 serving/d • Women aged 65 years and overs: <0.5 serving/d	3
	• Men aged 19-64 years: <0.5 serving/d • Women aged 19-64 years: <0.5 serving/d • Men aged 65 years and overs: <0.5 serving/d • Women aged 65 years and overs: <0.5 serving/d	0
Milk and milk products intake	≥1 serving/d	10
	0.5- <1 serving/d	5
	< 0.5 serving/d	0
Nuts	≥2 serving/week	5
	0 < 2 serving/week	3
	0 serving/week	0
Moderation		
Percentage of energy from saturated fatty acids	≤7% of total energy intake	10
	7<-9	7
	9<-11	4
	>11	0
Percentage of energy from polyunsaturated fatty acids	7<-9	10
	≥9	7
	≤7% of total energy intake	4
Sodium intake	≤2,000mg/d	10
	2000<-3000	7
	3000<-4000	4
	4000<	0
Percentage of energy from sweets and beverage	<10% of total energy intake	10
	10%-<-15% of total energy intake	7
	15%-<20% of total energy intake	4
	≥20% of total energy intake	0
Noodle intake	<0.5	5
	0.5-<1 serving/d	3
	≥1 serving/d	0
Beverage	0 serving	5
	0-0.7 serving/day for women, 0-1serving/day for men	3
	≥0.7serving/day for women, >=1 serving/d for men	0
Balance of nutrient intake		
Energy intake	75-120% of the estimated energy intake requirement (EER)	5
	65-75 or 120-135	3
	<65 or > 135	0
V-C intake	• Men aged 19-64 years: ≥ 100mg/d • Women aged 19-64 years: ≥100 mg/d • Men aged 65 years and overs: ≥100 mg /d • Women aged 65 years and overs: ≥100 mg /d	5
	• Men aged 19-64 years: 75<-100 mg/d • Women aged 19-64 years: 75<-100 mg /d • Men aged 65 years and overs: 75<-100 mg /d • Women aged 65 years and overs: 75<-100 mg /d	3
	• Men aged 19-64 years: <75 Women aged 19-64 years: <75mg/d • Men aged 65 years and overs: <75mg/d • Women aged 65 years and overs: <75mg/d	0
Fiber intake	• Men aged 19-64 years: ≥ 25g/d • Women aged 19-64 years: ≥20 g/d • Men aged 65 years and overs: ≥25 g /d • Women aged 65 years and overs: ≥20 g /d	5

	• Men aged 19-64 years: 15<-25g/d • Women aged 19-64 years: 10<-20 g /d • Men aged 65 years and overs: 15<-25 g /d • Women aged 65 years and overs: 10<-20 g /d	3
	• Men aged 19-64 years: <15 Women aged 19-64 years: <10g/d • Men aged 65 years and overs: <15g/d • Women aged 65 years and overs: <10g/d	0
Ca intake	• Men aged 19-64 years: ≥ 780 mg/d • Women aged 19-64 years: ≥730 mg/d • Men aged 65 years and overs: ≥700 mg /d • Women aged 65 years and overs: >=800 mg /d	5
	• Men aged 19-64 years: 630<-780 mg/d • Women aged 19-64 years: 540<-730 mg /d • Men aged 65 years and overs: 570<-700 mg /d • Women aged 65 years and overs: 560<-800 mg /d	3
	• Men aged 19-64 years: <630 Women aged 19-64 years: <540 g/d • Men aged 65 years and overs: <570mg/d • Women aged 65 years and overs: <560mg/d	0
Percentage of energy from carbohydrate	55-65% of total energy intake	5
	50-55 % or 65-70%	3
	<50 or >70	0
Percentage of energy intake from fat	15-30% of total energy intake	5
	10-15% or 30-35%	3
	<10 or >35	0

Fig. S1. Body mass index (BMI) and waist circumferences (WC) in the first recruitment according to gender and WC changes

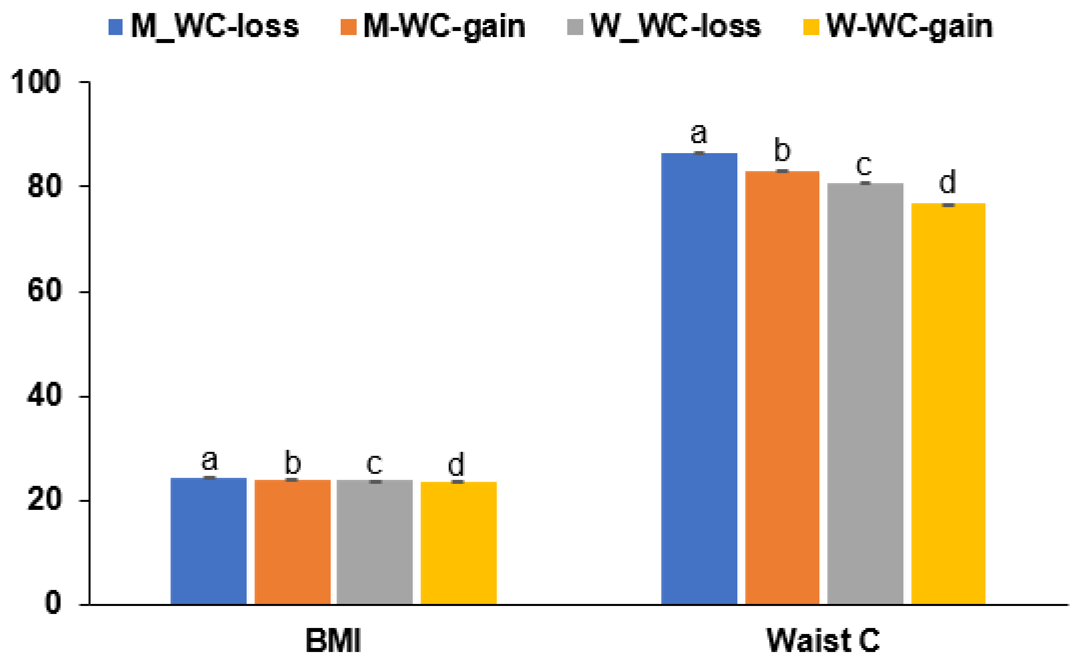

M, men; W, women. WC-loss, <0 in WC changes between the second and the first measurements; WC-gain, ≥ 0 in WC changes between the second and the first measurements.
